# Crucial Residues of C-Terminal Oligopeptide C60 to Improve the Yield of Prebiotic Xylooligosaccharides by Truncated Mutation

**DOI:** 10.3390/foods11060862

**Published:** 2022-03-18

**Authors:** Kungang Pan, Shanzheng Jin, Yue Wang, Zhao Yu, Junhao Sun, Tianhui Liu, Zhengjie Zhang, Tongcun Zhang, Zhongyuan Li, Junqi Zhao

**Affiliations:** 1 Qilu Institute of Technology, School of Chemical and Biological Engineering, Jinan 250200, China; pankungang@163.com; 2 Key Laboratory of Industrial Fermentation Microbiology of Ministry of Education & Tianjin Key Laboratory of Industrial Microbiology, College of Biotechnology, Tianjin University of Science and Technology, Tianjin 300457, China; oliverharrison@163.com (S.J.); wangyue11272022@163.com (Y.W.); yuzhao970426@163.com (Z.Y.); sjh1903834689@gmail.com (J.S.); liutianhui18@163.com (T.L.); zhangzhengjie2022@163.com (Z.Z.); tony@tust.edu.cn (T.Z.)

**Keywords:** prebiotic, xylooligosaccharides, xylanase, C-terminal region, crucial residues

## Abstract

Increasing the yields of short xylooligosaccharides by enzymatic production is efficient to improve prebiotic effects. Previously, C-terminal oligopeptide C60 was found to accelerate short xylooligosaccharides. Herein, in order to further understand the molecular mechanism of C60, the sequence analysis firstly showed that C60 displays typical properties of a linker (rich in proline/alanine/glycine/glutamine/arginine, 8.33–20.00%). C60 shared the highest identity with the N-terminal region of esterase (98.33%) and high identity with the linker between xylanase and esterase from *Prevotella* sp. (56.50%), it is speculated to originate from an early linker between XynA and another domain. Besides, structure simulation showed that C60 enhances the molecular interactions between substrate and active residues to improve catalytic efficiency. Moreover, three truncated variants with different lengths of C-terminal regions were successfully generated in *Escherichia coli*. The specific activities of variants were 6.44–10.24 fold of that of XynA-Tr, and their optimal temperature and pH were the same as XynA-Tr. Three truncated variants released more xylooligosaccharides, especially xylobiose (46.33, 43.41, and 49.60%), than XynA-Tr (32.43%). These results are helpful to understand the molecular mechanism of C60, and also provide new insight to improve the yields of short xylooligosaccharides by molecular modification at the terminal of xylanases.

## 1. Introduction

Prebiotic xylooligosaccharides (XOS) are sugar oligomers consisting of 2–7 xylosyl residues linked to each other by β-1,4 glycosidic bonds [1]. As a prebiotic, XOS can selectively promote the proliferation of probiotics like *Bifidobacteria* and *Lactobacillus* spp., thus significantly exhibiting a variety of health-benefiting effects such as promoting calcium absorption, lowering cholesterol, and reducing symptoms of diseases like diabetes, atherosclerosis, and colon cancer [2]. They also possess many biological activities including antimicrobial, antiallergy, and antioxidant activities. Compared with other functional oligosaccharides, XOS have good heat and pH stability, organoleptic properties, and price competitiveness [2]. Consequently, XOS are widely applied in various fields including food, cosmetics, and pharmaceuticals owing to their valuable traits, and the XOS global market is expanding at a high annual growth rate [3].

In view of the rising demand for XOS around the world, XOS are mainly produced from xylan in lignocellulosic biomass, since they exist in small amounts in fruits, vegetables, and dairy [4]. Compared with acid hydrolysis and auto-hydrolytic processes, enzymatic hydrolysis has particular advantages including less harsh conditions, higher efficiency and specificity, better control over the product’s degree of polymerization (DP), and more functional XOS yields [5]. In enzymatic hydrolysis, endo-β-1,4-xylanase (EC 3.2.1.8) is a rate-limiting enzyme, which randomly catalyzes the hydrolysis of β-1,4 xylose linkages in the backbone and yields XOS with various DP [6,7]. To date, endo-1,4-β-xylanase was classified into the glycoside hydrolases (GH) 5, 8, 10, 11, and 43 based on amino acid sequence homology comparison [8].

It has been shown that XOS with a low degree of polymerization such as xylobiose (X2) have enhanced prebiotic effects conferring health benefits to humans and animals [9]. Thus, increasing the yield of shorter XOS benefits promoting their prebiotic effects. Previously, the C-terminal oligopeptide C60 of GH10 xylanase XynA improved its catalytic efficiency by releasing more X2 [10]. It also exhibited similar effects when fused to other xylanases, suggesting its potential in the rational molecular design of xylanase to increase the yield of XOS. Terminal regions or residues have previously shown their significant effects on the physical and catalytic properties of enzymes such as thermostability and catalytic efficiency [11,12]. These effects of terminal regions or residues are related to their sequence length, amino acid type, and structure flexibility [13]. In this study, in order to understand and investigate the key factors contributing to the enhanced function of C60, three truncated mutants with different lengths of C-terminal regions were constructed on the basis of sequence analysis, and their effects on enzymatic properties and XOS production were comparatively investigated with the wild type.

## 2. Materials and Methods

### 2.1. Strains, Plasmids, and Chemicals

The recombinant strains pET-XynA and pET-XynA-Tr were constructed previously and used as the template in this study [10]. The expression vector pET-28a(+) was obtained from Invitrogen (Carlsbad, CA, USA). The stains of *E. coli* DH5α and BL21 (DE3) were cultured in Luria-Bertani (LB) medium (1% *w/v* tryptone, 0.5% *w/v* yeast extract, and 1% *w/v* NaCl) at 37 °C. Phusion DNA polymerase, restriction endonuclease, T4 DNA ligase, and alkaline phosphatase were purchased from ThermoFisher Scientific (Shanghai, China). A plasmid extraction kit and DNA purification kit were bought from Solarbio (Beijing, China). Xylan from beechwood used as xylanase substrate, xylooligosaccharide standards including xylobiose (X2), xylotriose (X3), xylotetraose (X4), xylopentaose (X5), and xylohexaose (X6) as well as xylose (X1) were all obtained from Sigma (St. Louis, MO, USA). All other analytical grade chemicals were commercially available.

### 2.2. Sequence and Protein Structure Analysis

The identities of XynA or C60 with other homologous sequences were analyzed by BLASTP programs (https://blast.ncbi.nlm.nih.gov/Blast.cgi, 1 February 2022). Multiple-protein sequence alignments were performed by ClustalW (http://www.ebi.ac.uk/clustalW, 15 January 2022). The amino acid contents of different sequences were calculated using Protein Stats (http://www.detaibio.com/sms2/protein_stats.html, 10 January 2022). The three-dimensional structural model of xylanases and C60 were predicted using SWISS-MODEL (https://swissmodel.expasy.org/, 4 March 2022) and the protein-structure prediction algorithm AlphaFold2 (https://github.com/deepmind/alphafoldRoseTTAFold, 4 March 2022). The protein structure and the molecular interaction were visualized by PyMOL software (https://pymol.org/2/, 4 March 2022). The active-site networks between XynA and X5 or X6 are viewed after superimposition of XynA with XOS from the xylanase complex structures (PDB code: 1r87 and 4pmd).

### 2.3. Construction, Protein Expression, and Purification of Truncated Variants

**Figure 1:**
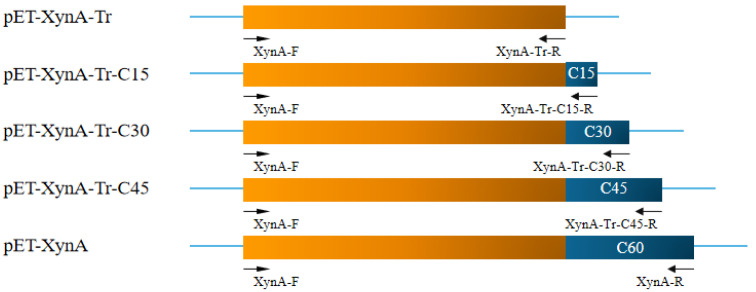
Schematic diagram of the construction of truncated mutants.

To investigate the effects of length of the C-terminal region on catalytic activity, three variants with different C-terminal peptides named XynA-Tr-C15, XynA-Tr-C30, and XynA-Tr-C45 were generated using the corresponding primer pairs, respectively (Figure 1 and Table S1). After being digested by *Eco*R I and *Not* I, these gene fragments of three variants were ligated into the pET-28a(+) vector and transformed into *E. coli* BL21 (DE3). The positive transformants harboring pET-XynA-Tr-C15, pET-XynA-Tr-C30, and pET-XynA-Tr-C45 were confirmed by DNA sequencing (GENEWIZ, Suzhou, China).

After induction by IPTG (1 mM) at 25 °C for 16 h, the recombinant proteins were eluted with 10 to 500 mM imidazole buffer (20 mM Tris-HCl, 50 mM NaCl, pH 8.0) using nickel-affinity chromatography (GE Health care, Uppsala, Sweden). The collected recombinant proteins were further concentrated by ultrafiltration and analyzed by sodium dodecyl sulfate (SDS)-polyacrylamide gel electrophoresis (PAGE). A protein assay kit was used to determine the protein concentration (Bio-Rad, Hercules, CA, USA).

### 2.4. Enzymatic Activity Assay and Characterization

A 3,5-dinitrosalicylic acid (DNS) assay was applied to determine xylanase activity by measuring the release of reducing sugar [14]. A total of 950 μL beechwood xylan substrate (1%, *w*/*v*) and 50 μL protein sample (2 μM) were mixed in each reaction system. 

To evaluate the effects of different C-terminal peptides on catalytic activities, the optimum temperature of all variants was determined at different temperatures of 30–60 °C at pH 6.0, and the optimal pH was measured using 0.2 M citric acid-Na2HPO4 buffer (pH 4.0–8.0) at the optimum temperature. A total of 1 unit (U) is the amount of xylanase needed to release 1 μmol of reducing sugar per minute at optimal reaction conditions (pH 6.0 and 45 °C). The increased folds of catalytic activity of the variants were calculated using the specific activity of XynA-Tr as control.

### 2.5. XOS Production Analyzed by High-Performance Anion-Exchange Chromatography 

Each reaction system contained 900 μL of 0.5% (wt/vol) beechwood xylan and 100 μL of each enzyme sample (10 U of XynA, XynA-Tr, XynA-Tr-C15, XynA-Tr-C30, or XynA-Tr-C45) and was incubated at optimal conditions. After 12 h, the residual enzymes were removed from the reaction system using Nanosep^®®^ centrifugal 3K devices (Pall, New York, NJ). The released products of XynA, XynA-Tr, XynA-Tr-C15, XynA-Tr-C30, and XynA-Tr-C45 were detected by high-performance anion-exchange chromatography (HPAEC) eluted by buffer (100 mM NaOH, 210 mM sodium acetate). A Dionex CarboPac PA100 (4 by 250 mm) column (Sunnyvale, CA, USA) and pulsed amperometric detection (PAD) were used. To identify hydrolysis products, xylose (X1), xylobiose (X2), xylotriose (X3), xylotetraose (X4), xylopentaose (X5), and xylohexaose (X6) were used as controls.

### 2.6. Statistical Analysis 

In this study, the experiments were repeated three times, and the data were plotted using the SD (mean ± standard deviation) method using Graphpad Prism 7 software (San Diego, CA, USA). Significance analysis was performed by t-test at a significance level of 0.05% using Prism 7.

## 3. Results and Discussions

### 3.1. Sequence Alignment and Protein Structure Analysis of C-Terminal Oligopeptide C60

Our previous study reported that XynA, a predominant xylanase in a rumen competitive environment, contains a catalytic domain XynA-Tr and a C-terminal oligopeptide C60. C60 improved the hydrolysis efficiency of the catalytic domain by increasing the yields of xylobiose and xylose [15]. Homology modeling indicated that the three-dimensional structural model of the catalytic domain (XynA-Tr) displays a typical GH10 family protein structure. GH10 family xylanase is one of the most employed xylanases for XOS production due to their high substrate specificity, efficiency, and yield of XOS [16,17,18]. 

**Figure 2:**
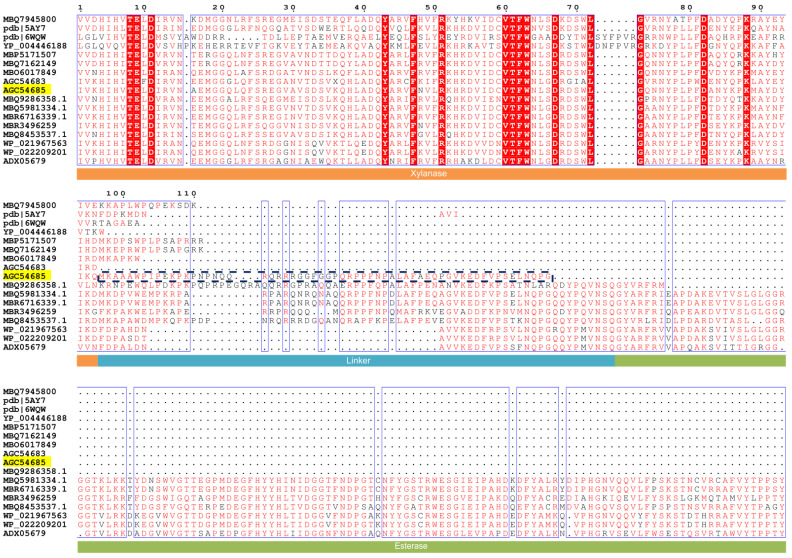
The amino acid sequence alignment regarding XynA and C-terminal region C60 with the other homologous sequences. The sequences contain the xylanases from *Bacteroidales* bacterium (MBQ7945800, MBP5171507, MBQ7162149, and MBQ9286358.1), *Aegilops speltoides sub* sp. *speltoides* (pdb|5AY7), *Thermobacillus* (pdb|6WQW), *Haliscomenobacter hydrossis* (YP_004446188), *Prevotella* sp. (MBQ5981334.1, MBR6716339.1, MBR3496259, and MBQ8453537.1), *Bacteroides intestinalis* (WP_021967563), and *Bacteroides cellulosilyticus* (WP_022209201). The same amino acids are marked in dark red and red. The bottom orange rectangle is the xylanase domain, the blue rectangle is the linker region, and the green rectangle is the esterase domain. The dark blue dashed box marked in the sequence is C60.

In this study, the key amino acid residues affecting C60 function were further investigated. Firstly, using an intact amino acid sequence of XynA as the query sequence, XynA-Tr exhibited above 80% sequence identity with endo-1,4-beta-xylanase from *Prevotella* sp. (GenBank accession number MBQ5981334), whereas the homologous sequences of C60 were not found in alignments by BLASTP analysis. Thereby, the 60 residues of C60 were aligned individually, and the results suggested that it shows the highest identity (98.33%) with the N-terminal region of an esterase from *Prevotella* sp. (GenBank accession No. MBQ6229348), which is annotated based on DNA sequencing from the ruminant gastrointestinal microbiome. It also shared a high identity (56.50%) with the linker region between xylanase and esterase from *Prevotella* sp. Besides, it is worth noting that the majority of xylanases sharing high identity with XynA-Tr were followed by another domain of enzymes like acetylxylan esterase (Figure 2), such as xylanases from *Prevotella* sp. (MBQ5981334.1, MBR6716339.1), *Bacteroides intestinalis* (WP_021967563), and *Bacteroides cellulosilyticus* (WP_022209201). Acetylxylan esterase (EC 3.1.1.72) is an important enzyme for the hydrolysis of side chains in xylan degradation [19]. Usually, several functionally related proteins such as xylanase and ersterase are encoded in a single mRNA strand named polycistron, which is very common in prokaryotes. Between these domains in a gene cluster there are some short amino acid sequences called linkers, which are responsible for connecting various domains without interfering with their function [20]. As shown in sequence alignment (Figure 2), C60 matched the corresponding position of linkers between the xylanase domain and the following esterase domain from *Prevotella* sp. (MBQ5981334, MBR6716339, MBR3496259, and MBQ8453537), *Bacteroides intestinalis* (WP_021967563), and *Bacteroides cellulosilyticus* (WP_022209201, ADX05679). The sequence length of these linkers in sequence alignment varies from 30 to 66 residues, which are similar to that of C60. Among these linkers, BLASTP analysis found that the amino acid sequence of C60 shares the highest identity (56.50%) with the linker sequence from *Prevotella* sp. (MBQ8453537).

**Figure 3:**
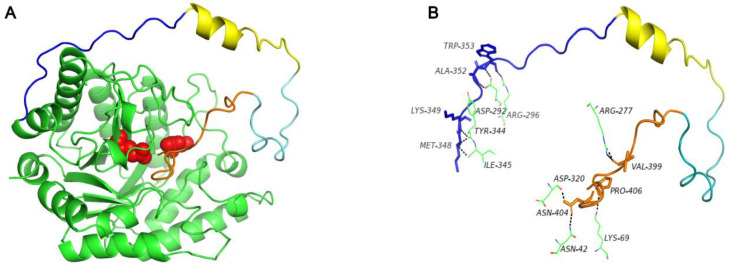
Three-dimensional protein structure model of XynA (**A**) and the molecular interactions between C60 and XynA-Tr (**B**). XynA-Tr is colored in green, the four parts of C60 are marked in dark blue, yellow, light blue, and orange, respectively. The black dashed line represents the molecular interactions.

**Figure 4:**
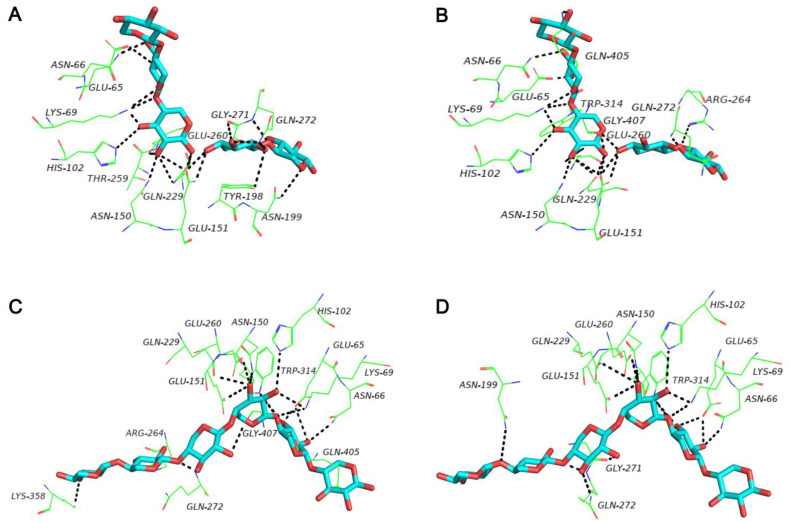
Detailed interaction networks in the active site of XynA with X5 (**A**), XynA-Tr with X5 (**B**), XynA with X6 (**C**), and XynA-Tr with X6 (**D**) after superimposition. The residues of XynA and XynA-Tr that interacted with X5 or X6 are shown as green lines, X5 and X6 are shown with blue sticks. Black dashed lines represent the hydrogen bonds.

Using SWISS-MODEL software, we failed to obtain the protein structure of C60, because no matched templates were found for the target sequence. However, the three-dimensional structure of C60 was successfully predicted by Alphafold2. C60 consisted of loops and a short helix region (Figure 3), and there are 11 molecular interactions formed between C60 and XynA-Tr (Figure 3). Besides, as shown in Figure 4, the predicted structure of XynA and XynA-Tr were superimposed onto the xylanase-X5 structure (PDB code: 1R87) from the *Geobacillus stearothermophilus* and xylanase-X6 structure (PDB code: 4pmd) from *Caldicellulosiruptor bescii*, respectively. For X5, 21 hydrogen bonds were formed in the active-site networks of XynA (Figure 4A), while 20 hydrogen bonds were seen in XynA-Tr (Figure 4B). In terms of X6, there are 17 hydrogen bonds and 16 hydrogen bonds found in XynA and XynA-Tr (Figure 4C,D), respectively. The increase of hydrogen bonds in XynA might result in a higher affinity between active residues and substrate, which is in correspondence with previous ITC analysis [10]. 

### 3.2. Residue Contents Analysis of C-Terminal Oligopeptide C60

The amino acid ratios of XynA-Tr, C60, and other linkers in the sequence alignment were comparatively analyzed (Table 1). Compared with XynA-Tr, several typical linker-related residues such as proline (P), alanine (A), glycine (G), glutamine (Q), and arginine (R) were all rich in the amino acid sequence of C60. The ratio of proline in C60 was the highest (20.00%), followed by alanine (10.00%), glycine (10.00%), glutamine (10.00%), and arginine (8.33%). Similar to C60, the ratios of P, A, Q, and R residues were also abundant in other linker regions. Prolines, the richest residues of C60, are often found in linker peptides between natural multi-structural domain proteins, and they have been shown to have significant resistance to degradation by proteases. Because the side chain of the proline has a typical cyclic formation and locks its backbone, the proline-rich sequence was recognized to rigidify the main chain and thus improve its thermostability [21]. Interestingly, arginine often appears in the proline-rich linker, which has a stronger ability to form hydrogen bonds and salt bridges [22]. Thus, the enriched proline and arginine residues of C60 might result in the better thermostability of XynA compared to XynA-Tr, mentioned previously [23]. Glycine is usually involved in flexible linkers, and the “GGGGS” motif is a common flexible unit that appears in linkers. These peptides increase proteins solubility and provide the flexibility required by proteins in the catalytic process since they do not have the ability to form a specific secondary structure [20].

**Figure 5:**
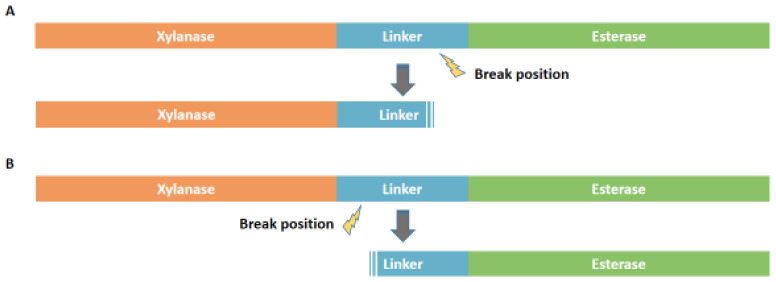
Schematic diagram of the formation of C60. (**A**) The linker located at the C-terminal of xylanase after breaking. (**B**) The linker located at the N-terminal of esterase after breaking.

Thus, in combination with peptide length, residues ratio, and sequence alignment, it is speculated that C60 might previously originate from the early linker region between XynA-Tr and the following acetylxylan esterase. However, the gene clusters were broken randomly at different positions of the linker region during the natural evolution process, thus forming XynA with C-terminal peptides (Figure 5A) and esterase with N-terminal peptides from *Prevotella* sp. (GenBank accession number MBQ6229348) (Figure 5B), respectively. 

### 3.3. Enzyme Expression, Purification, and Characterization of Truncated Variants

Utilizing protein engineering is an effective strategy for improving enzyme characteristics such as specific activity and stability. Generally, functional regions like the catalytic site are usually recognized as the typical target regions to modify wild-type enzymes. In addition to the catalytic domain, it has been well documented that N- and C-terminal regions are also crucial factors for enzyme properties [13]. For example, proline-rich sequence broadens the optimal pH and temperature ranges of xylanase from *Geobacillus thermodenitrificans* C5 [23], the chaperone-like ability of artemin is reduced by deletion of its extra C-terminal 39 residues [24], and the thermostability and optimal temperature of GH11 xylanase increased after C-terminal region deletion [25]. 

**Figure 6:**
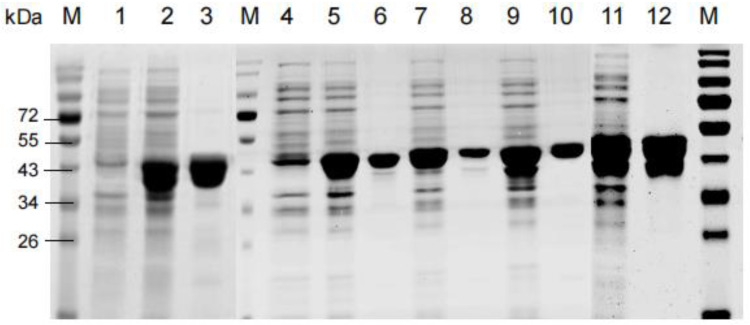
SDS-PAGE of purified recombinant proteins of XynA and its variants. Lane M: molecular weight markers; lane 1 and 4: culture supernatant of pET-28a(+) in *E. coil* BL21; lane 2: culture supernatant of XynA-Tr; lane 3: purified XynA-Tr; lane 5: culture supernatant of XynA-Tr-C15; lane 6: purified XynA-Tr-C15; lane 7: culture supernatant of XynA-Tr-C30; lane 8: purified XynA-Tr-C30; lane 9: culture supernatant of XynA-Tr-C45; lane 10: purified XynA-Tr-C45; lane 11: culture supernatant of XynA; lane 12: purified XynA.

Generally, the function of linkers is determined by many factors such as its sequence length and critical residues types [13,20]. As shown in sequence alignment (Figure 2), the first 15 residues of C60 showed high identity with the C-terminal sequences of xylanases with a single domain (MBQ5171507 and MBQ7162149). In order to investigate the key residues on the effects of C60, it was divided into 4 parts and each part contained 15 residues. The variants named XynA-Tr-C15, XynA-Tr-C30, and XynA-Tr-C45 were successfully amplified, ligated into pET28a(+), and transformed into *E. coli*. After induction by IPTG for 12 h, the crude proteins of positive transformants showed xylanase activities. All purified recombinants of XynA-Tr-C15, XynA-Tr-C30, and XynA-Tr-C45 showed a single band of 39.09, 40.66, and 42.45 kDa by protein SDS-PAGE analysis (Figure 6), respectively, which are identical to their theoretical molecular weight values.

**Figure 7:**
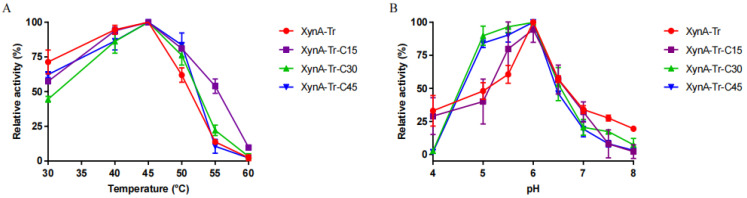
Effects of temperature (**A**) and pH (**B**) on the activity of the variants.

**Figure 8:**
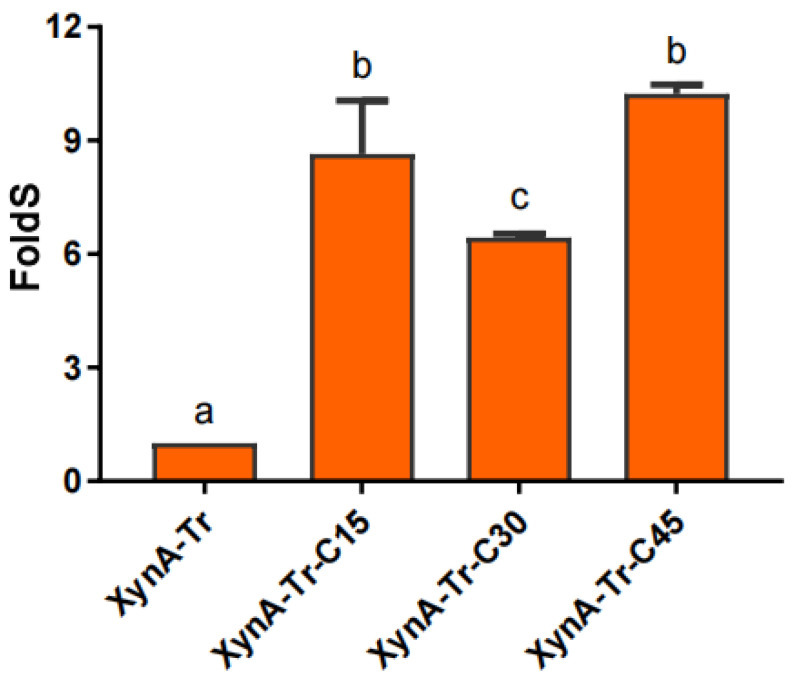
The specific activities of the variants. Results are expressed as means of three replicates and standard errors. Means with different letters are significantly different according to *t*-tests (*p* < 0.05).

Similar to XynA-Tr, the optimal temperature of XynA-Tr-C15, XynA-Tr-C30, and XynA-Tr-C45 were still 45 °C (Figure 7). Besides, three variants had similar pH optima of 6.0, which is identical to that of XynA-Tr. XynA-Tr-C30 and XynA-Tr-C45 showed higher relative activity at acidic conditions (pH 5.0 and pH 5.5) (Figure 7). These results indicated that three C-terminal regions with different lengths do not affect the optimal temperature and pH, which allows these variants to remain active at a wide range of pHs and temperatures. Besides, the specific activities of XynA-Tr-C15, XynA-Tr-C30, and XynA-Tr-C45 were 8.65, 6.44, and 11.24 fold of that of XynA-Tr (Figure 8), which is similar to that of C60 [10]. There was no significant difference between XynA-Tr-C15 and XynA-Tr-C45 by t-test analysis. In consideration of atom economy, increasing the catalytic efficiency by fusing with C15 is a preference for molecular modification. The lower specific activity of XynA-Tr-C30 is might due to the unique helix secondary structure in the C30 region (Figure 3), which is needed to be studied in further research. 

### 3.4. Analysis of XOS Production of Truncated Variants

The hydrolysis products of XynA-Tr, XynA-Tr-C15, XynA-Tr-C30, XynA-Tr-C45, and XynA against beechwood xylan were simultaneously analyzed (Table 2). All these truncated variants and the wild type produced a large amount of XOS including X2, X3, and X4 (75.22–80.78%), and a small quantity of X1 (8.75–14.43%). Among these XOS, X2 was the main hydrolysis product, followed by X3. It has been well documented that degraded products of xylan by different xylanases are diverse, which is not only dependent on the family but also on individual genes. Some GH10 xylanases mainly produce X1 and X2 [26], while the hydrolysis products of some xylanases were dominated with a higher DP [27].

Besides, compared with XynA-Tr, the total ratio of producing XOS (X2, X3, and X4) of variants XynA-Tr-C15, XynA-Tr-C30, and XynA-Tr-C45 were obviously enhanced. In particular, the ratios of producing X2 significantly increased to 46.33, 43.41, 49.60, and 45.82% from 32.43% (XynA-Tr), respectively. These products have a similar constitution to C15, C30, C45, and C60, clearly suggesting the first 15 residues are crucial to promoting xylan degradation, which is in accordance with the improvement of a specific activity. It is well known that XOS with a lower DP shows a higher prebiotic potential, and X2 has been reported to have the strongest prebiotic effects among XOS [3]. In consideration of its lower sweetness and energy than sucrose, X2 exhibited high marker interest as a health-promoting bulk sweetener ingredient [28]. Thus, increasing the yields of shorter XOS especially X2 by enzymatic production is efficient to improve their prebiotic effects. In this study, the improvement of produced X2 by fusing C-terminal regions clearly suggested the applicability of genetic modification such as the introduction of C15 to increase the yield of XOS. 

Moreover, C-terminal regions were reported to be involved in the enzyme–substrate reaction, like the C-terminus segment of phospholipase A2 changes the substrate specificity and substrate binding [29]. Since the distance between the C-terminal end and the catalytic residues of XynA-Tr is far (above 24 Å), the improved effects of C60 might be related to remote influence on substrate binding, which is needed to be studied in the future.

## 4. Conclusions

In the present study, it was found that the C-terminal region C60 exhibits typical characteristics of the linker region, improves the catalytic efficiency, and releases more XOS through increasing the molecular interaction between substrate and active residues. The critical residues of C60 were also identified by mutation analysis. This study provides new insight into the molecular mechanism of C60 and offers an efficient strategy to improve XOS production by molecular modification.

## Figures and Tables

**Table 1:** Comparison of amino acid contents of different linkers.

Gene	Proline (P)		Alanine (A)		Glycine (G)		Glutamine (Q)		Arginine (R)		Glutamic Acid (E)		Phenylalanine (F)		Lysine (K)		Asparagine (N)	
	N	%	N	%	N	%	N	%	N	%	N	%	N	%	N	%	N	%
XynA-Tr	15	4.34	30	8.67	16	4.62	17	4.91	17	4.91	20	5.78	15	4.34	24	6.94	22	6.36
C60	12	20	6	10	6	10	6	10	5	8.33	4	6.67	4	6.67	4	6.67	4	6.67
MBQ5981334	11	17.46	5	7.94	2	3.17	10	15.87	5	7.94	4	6.35	3	4.76	3	4.76	5	7.94
MBR6716339	11	17.46	5	7.94	2	3.17	10	15.87	5	7.94	4	6.35	3	4.76	3	4.76	5	7.94
MBR3496259	10	16.67	4	6.67	2	3.33	9	15	5	8.33	3	5	4	6.67	5	8.33	4	6.67
MBQ8453537	11	16.67	5	7.58	3	4.55	9	13.64	5	7.58	4	6.06	3	4.55	6	9.09	4	6.06
MBQ9286358	13	17.81	8	10.96	2	2.74	11	15.07	8	10.96	5	6.85	3	4.11	5	6.85	5	6.85

N represents the number of each residue; % represents the percentage of each residue.

**Table 2:** Products of xylan degraded by five xylanases.

Hydrolysis Products	% Hydrolysis Products ^a^				
	XynA-Tr	XynA-Tr-C15	XynA-Tr-C30	XynA-Tr-C45	XynA
Xylose	11.98	8.25	13.24	8.75	14.43
Xylobiose	32.43	46.33	43.41	49.60	45.82
Xylotriose	38.00	32.36	30.41	30.80	32.45
Xylotetraose	5.37	1.39	1.40	0.37	2.03
Xylobiose + Xylotriose + Xylotetraose	75.80	80.08	75.22	80.78	80.31
Other xylooligosaccharides	12.22	11.67	11.54	10.48	5.26

^a^ The total amount of all detected products is defined as 100%, and the amount of each hydrolysis product is shown by the percentage of the total amount; the detection limit was 0.01%.

## Data Availability

The data presented in this study are available within the article.

## Supplementary Materials

The following supporting information can be downloaded at: https://www.mdpi.com/article/10.3390/foods11060862/s1, Table S1: The primers used in this study.
