# Supplementary material for: Crucial Residues of C-Terminal Oligopeptide C60 to Improve the Yield of Prebiotic Xylooligosaccharides by Truncated Mutation
Source: Foods. 2022 Mar 18;11(6):862. doi: 10.3390/foods11060862 (PMC8954191; doi:10.3390/foods11060862)
Supplement: Supplementary file 1 [file foods-11-00862-s001.zip › foods-1618651-supplementary.pdf]

Table S1. primers sequence used in this study.

Primers	Sequence (5'→3')
XynA-F	GGG GAA TTC CAA GGC CTG AAA GAC ATC TAC AAG GAC TAC TTC
XynA-Tr-R	GGG GCG GCC GCG CTG CTT GAT ATA GTC GTA GGC CAT CTT GGG
XynA-Tr-C15-R	GGG GCG GCC GCG GTT GGG TTT CGG TTT CTC GGG GAT GG
XynA-Tr-C30-R	GGG GCG GCC GCG TGG ACC GCC AAA TCC GCC ACG AC
XynA-Tr-C45-R	GGG GCG GCC GCG AGG TTG CTC GGC GAA AGC CAG TGC
XynA-R	GGG GCG GCC GCG TCC AGG CTG GTT GAG CTC AGA GGG
